# Supplementary material for: The Effect of Hypothermia and Osmotic Shock on the Electrocardiogram of Adult Zebrafish
Source: Biology (Basel). 2022 Apr 15;11(4):603. doi: 10.3390/biology11040603 (PMC9025276; doi:10.3390/biology11040603)
Supplement: Supplementary file 1 [file biology-11-00603-s001.zip › biology-1603121-supplementary.pdf]

biology

MDPI

Supplementary Materials

The Effect of Hypothermia and Osmotic Shock on the Electrocardiogram of Adult Zebrafish

Elodie Arel [1,2,†], Laura Rolland [2,†], Jérôme Thireau [1], Angelo Giovanni Torrente [2], Emilie Bechard [1], Jamie Bride [1,2], Chris Jopling [2,*], Marie Demion [1,‡] and Jean-Yves Le Guennec [1,‡]

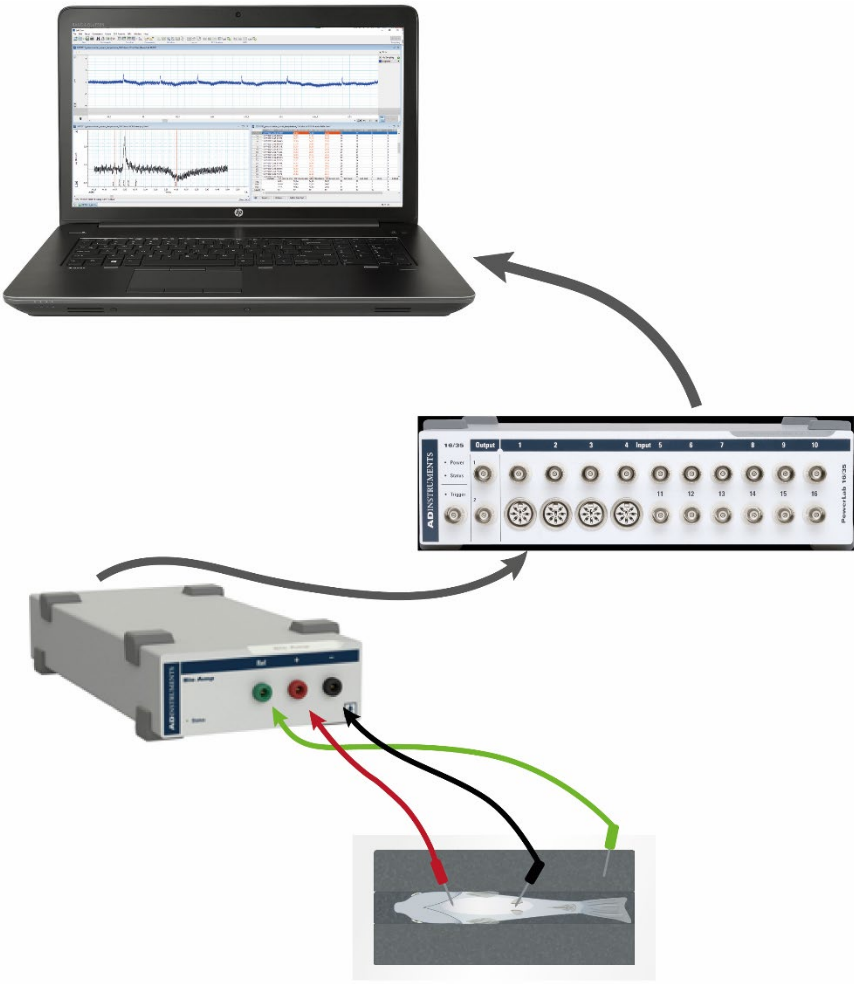

Figure S1. ECG recording setup. Anesthetized zebrafish were placed ventral side up in a slit sponge. Two 29-gauge stainless steel micro-electrodes (MLA1213, AD Instruments) were positioned along the ventral midline as shown. The reference electrode (green electrode) was either placed in the bath or out, depending of what created the clearest signal. ECG signals were amplified and digitized using a BioAmp (FE231, AD Instruments) and a PowerLab (16/35, AD Instruments). Data were subsequently processed using LabChart Pro v8 Software and the ECG analysis module (AD Instrument). Recordings were made in the range 0-10mV. A 50Hz notch digital filter was then applied and a sliding averaging algorithm provided by the software was used to smooth the traces.

Table S1. Osmotic shock P and T wave detection. Proportion of ECG complexes presenting P/T waves during a 1-minute recording. Data are presented as mean (SEM). The sample size (n) is indicated in brackets for each parameter in the table. NC: not calculated.

Configuration Condition	P wave (%)	T wave (%)
**Unexposed heart**		
**Tyrode**	94.1% (1.9) *(10)*	45.6% (14.7) *(5)*
**Isosmotic**	78.4% (8.7) *(9)*	56.4% (18.0) *(5)*
**Hyposmotic**	76.2% (10.5) *(9)*	40.4% (16.2) *(5)*
**Wash isosmotic**	79.0% (10.0) *(7)*	26.7% (17.4) *(3)*
**Hyperosmotic**	79.0% (8.8) *(8)*	39.8% (17.9) *(4)*
**Exposed heart**		
**Tyrode**	91.2% (8.5) *(10)*	90.8% (3.2) *(10)*
**Isosmotic**	98.3% (0.7) *(9)*	86.9% (4.4) *(8)*
**Hyposmotic**	98.8% (0.5) *(9)*	84.0% (7.2) *(6)*
**Wash isosmotic**	98.2% (1.2) *(9)*	82.2% (6.5) *(6)*
**Hyperosmotic**	99.7% (0.2) *(9)*	92.6% (2.5) *(5)*
**Extracted heart**		
**Tyrode**	NC (NC) *(0)*	60.1% (15.3) *(7)*
**Isosmotic**	NC (NC) *(0)*	73.5% (10.5) *(8)*
**Hyposmotic**	NC (NC) *(0)*	86.6% (4.0) *(7)*
**Wash isosmotic**	NC (NC) *(0)*	77.1% (11.6) *(8)*
**Hyperosmotic**	NC (NC) *(0)*	92.1% (5.0) *(7)*

Table S2. Hypothermia P and T wave detection. Proportion of ECG complexes presenting P/T waves during a 1-minute recording. Data are presented as mean (SEM). The sample size (n) is indicated in brackets for each parameter in the table. NC: not calculated.

Configuration Condition	P wave (%)	T wave (%)
**Unexposed heart**		
**Warm (control)**	86.4% (6.7)<br>*(10)*	62.6% (8.8)<br>*(5)*
**Cold**	59.4% (10.5)<br>*(10)*	NC (NC)<br>*(1)*
**Warm (washout)**	68.2% (10.6)<br>*(9)*	17.3% (0.7)<br>*(3)*
**Exposed heart**		
**Warm (control)**	75.4% (32.8)<br>*(8)*	96.5% (1.6)<br>*(6)*
**Cold**	80.7% (7.9)<br>*(9)*	71.9% (7.3)<br>*(9)*
**Warm (washout)**	91.8% (3.1)<br>*(9)*	82.5% (5.9)<br>*(8)*
**Extracted heart**		
**Warm (control)**	62.0% (14.9)<br>*(7)*	61.3% (9.6)<br>*(8)*
**Cold**	85.0% (10.2)<br>*(4)*	68.2% (13.4)<br>*(6)*
**Warm (washout)**	59.5% (22.5)<br>*(4)*	58.4% (11.2)<br>*(7)*
